# Development and Application of a Duplex RT-RPA Assay for the Simultaneous Detection of *Cymbidium mosaic virus* and *Odontoglossum ringspot virus*

**DOI:** 10.3390/v16040543

**Published:** 2024-03-30

**Authors:** Aiqing Sun, Lihua Wang, Yiping Zhang, Xiumei Yang, Yan Su, Xuewei Wu

**Affiliations:** 1 Yunnan Provincial Key Laboratory of Flower Breeding, Flower Research Institute, Yunnan Academy of Agriculture Science, Panlong District, Kunming 650025, China; sunaiqing@mail.ynu.edu.cn (A.S.); blackfarinj@126.com (Y.Z.); yangxm77@sina.com.cn (X.Y.); wlh25252024@163.com (Y.S.); 2 School of Agriculture, Yunnan University, Chenggong District, Kunming 650091, China

**Keywords:** *Cymbidium mosaic virus* (CymMV), *Odontoglossum ringspot virus* (ORSV), reverse transcriptase recombinase polymerase amplification (RT-RPA), simultaneous detection, orchid, plant diagnosis

## Abstract

*Cymbidium mosaic virus* (CymMV) and *Odontoglossum ringspot virus* (ORSV) are among the world’s most serious and widespread orchid viruses; they often infect orchids, causing devastating losses to the orchid industry. Therefore, it is critical to establish a method that can rapidly and accurately detect viruses in the field using simple instruments, which will largely reduce the further spread of viruses and improve the quality of the orchid industry and is suitable for mass promotion and application at grassroots agrotechnical service points. In this investigation, we established a rapid amplification method for virus detection at 39 °C for 35 min to detect the presence of CymMV and ORSV simultaneously, sensitively, and specifically in orchids. Primers for the capsid protein (CP)-encoding genes of both viruses were designed and screened, and the reaction conditions were optimized. The experimental amplification process was completed in just 35 min at 39 °C. There were no instances of nonspecific amplification observed when nine other viruses were present. The RPA approach had detection limits of 10^4^ and 10^3^ copies for pMD19T-CymMV and pMD19T-ORSV, respectively. Moreover, the duplex RT-RPA investigation confirmed sensitivity and accuracy via a comparison of detection results from 20 field samples with those of a gene chip. This study presents a precise and reliable detection method for CymMV and ORSV using RT-RPA. The results demonstrate the potential of this method for rapid virus detection. It is evident that this method could have practical applications in virus detection processes.

## 1. Introduction

Orchidaceae is one of the largest families of flowering plants, comprising over 28,000 species across more than 800 genera [1]. Orchids, as important potted and cut flowers worldwide, make up 1/10 of the global flower trade [2]. Infection from over 50 viruses has been identified in orchids around the world, including *Tospoviruses*, *Potexviruses*, and *Tobamoviruses* [3,4,5,6]. *Cymbidium mosaic virus* (CymMV) and *Odontoglossum ringspot virus* (ORSV) are two of the most prevalent and destructive viruses detected in orchids. These viruses have been identified and reported in numerous countries, including China, Indonesia, Mexico, South Korea, and Vietnam [7,8,9,10,11]. CymMV belongs to *Potexviruses*, which have linear virions. Orchids infected with CymMV show symptoms such as etiolation, necrosis, ring spots, and teratogeny [12]. The main ways of transmission are aphid transmission and mechanical contact infection, and they can also be transmitted by seeds [13]. ORSV belongs to *Tobamoviruses*, which have virus particles that are straight rod-shaped. The symptoms after infection with ORSV are embedded stripes, mottled stripes, yellowing stripes, ring spots, and floral streaks. Contact causes micro wounds, which is the main method of *Tobamovirus* transmission. Thus, via direct contact with the infected sap, the use of contaminated equipment by the diseased plant may transmit the virus [12]. Orchid viruses commonly cause complex infections in plants, leading to a natural field infection rate of over 30%. This results in significant losses to the ornamental and economic value of orchids. Infected plants display symptoms such as leaf necrosis or necrotic spots, abnormal growth, petal discoloration, and plant dwarfism, which significantly reduce their vitality. Both CymMV and ORSV can be transmitted via juice, aphids, and contact. Viruses are commonly referred to as “plant cancers” due to their harmful effects on plants [14]. Unfortunately, there is currently no specific cure for the prevention and control of these viruses. Therefore, strengthening quarantine procedures and timely destruction of infected plants have proven to be effective measures in reducing the occurrence and harm of orchid virus diseases [7,15,16].

Numerous approaches for detecting CymMV and ORSV have been documented thus far [1,14,17,18]. Enzyme-linked immunosorbent assay (ELISA), reverse transcription polymerase chain reaction (RT-PCR), reverse transcription loop-mediated isothermal amplification (RT-LAMP), and quantitative RT-PCR (qRT-PCR) are among those methods. Nevertheless, these methods confront technical restrictions [19]. For instance, virus detection via ELISA relies on high-quality and valid antibodies, which incurs significant cost and time expenditure [20]. Moreover, RT-PCR is a technology that necessitates specific operational proficiency and can be time-consuming. RT-LAMP is an isothermal amplification technique. However, its application is restricted, as it necessitates the design of four or more primers, which is a complicated process [21]. qRT-PCR requires expensive thermocyclers and real-time fluorescence data reading instruments [19,22].

With the advancement of isothermal amplification technology, Piepenburg and colleagues developed recombinase polymerase amplification (RPA) in 2006 [23]. RPA offers benefits such as high sensitivity, fast amplification, and time-efficient and convenient operation [19,23,24]. The RPA reaction operates isothermally by using enzymes to separate the double-stranded DNA rather than denaturing the target DNA strand with heat [25]. After incubation at 37–42 °C for 40 min, the amplified products can be detected [26]. Agarose gel electrophoresis, real-time fluorescent probes, and lateral flow immunoassay are among the methods used to visualize the amplification products of RPA [27]. In recent years, RT-RPA technology has been utilized for plant virus detection, including but not limited to *barley yellow dwarf virus* [28], *cucurbit chlorotic yellows virus* [29], *apple chlorotic leaf spot virus* [30], *potato virus Y* [31], and *chilli veinal mottle virus* [32]. When compared to uniplex RPA detection, multiple RPA detection proves to be more cost-effective, time-saving, and efficient [33]. The initial report on the multiplex RPA assay pertained to the concurrent identification of three bacterial pathogens [34]. Additionally, a study outlined the creation of a multiplex lateral flow detection RPA assay for the detection of up to seven DNA targets in a single RPA reaction [35,36]. Various other investigations have documented the development of multiplex RPA assays for the detection of pathogens in plant, animal, and human DNA samples [37,38].

To date, no study has documented the creation of an RPA approach for spotting CymMV and ORSV. Here, we present a straightforward, effective RPA test that can detect both CymMV and ORSV simultaneously. The assay provides technical assistance for field identification and risk estimation of orchids. Additionally, we validated the method using naturally infected samples from diseased fields. We expect that this method will provide technical support for the diagnosis, monitoring, and identification of orchid virus diseases of grassroots agrotechnical service points.

## 2. Materials and Methods

### 2.1. Virus and Plant Material

#### 2.1.1. Positive Samples

Samples positive for viruses were procured from Agdia, Inc. in the United States and Bulb Quality Support B.V. in the Netherlands. The Agdia Catalog numbers for different viruses are as follows: LPC89501 (CymMV), LPC54301 (ORSV), LPC33001 (*cymbidium ringspot virus*, CymRSV), LPC45702 (*cucumber green mottle mosaic virus*, CGMMV), LPC57400 (*tobacco mosaic virus*, TMV), SRA91500 (*plantago asiatica mosaic virus*, PLAMV), LPC10000 (*potato virus X*, PVX), LPC45400 (*tomato bushy stunt virus*, ToBSV), LPC44501 (*cucumber mosaic virus*, CMV), LPC68000 (*carnation mottle virus*, CarMV). The Bulb Quality Support Catalog number for *lily mottle virus* (LMoV) is LMoV-pos.

#### 2.1.2. Samples Collected from the Field for Detecting

From 2021 to 2023, 20 orchids with signs suggestive of viral infection, such as necrotic leaf spots, chlorosis, leaf deformation or roughness, were collected from growing areas of Yunnan, Guangxi, Guangdong, and Fujian provinces in the People’s Republic of China, which including 2 cultivars of *Dendrobium*, 3 cultivars of *Oncidium*, 5 cultivars of *Zygopetulum*, and 10 cultivars of *Phalaenopsis*, and their leaves were cut and cryopreservation at −80 °C.

### 2.2. Total RNA Extraction and Reverse Transcription

RNA was extracted from a leaf sample using the TaKaRa MiniBEST Plant RNA Extraction Kit (Code 9679) (Takara Biomedical Technology (Beijing) Co., Ltd., Beijing, China) in accordance with the manufacturer’s instructions. Following the same instructions from the TaKaRa PrimeScript™ II 1st Strand cDNA Synthesis Kit (Code 6210) (Takara Biomedical Technology (Beijing) Co., Ltd., Beijing, China), first-strand cDNA was synthesized by reverse transcription reaction using Oligo dT Primer and Random 6 mers as primers, PrimeScript II RTase as reverse transcriptase. The quality and quantity of the extracted nucleic acid were evaluated using a NanoDrop 2000 spectrophotometer (Thermo Fisher Scientific, Waltham, MA, USA). The RNA was stored at −80 °C, and the cDNA was stored at −20 °C until further use. The product was subsequently employed as a template for RPA.

### 2.3. Primer Design and Synthesis

Ten CymMV conserved gene sequences, including NP054029.1, AB693982.1, AM236030.1, MG774930.1, and so on, fifteen ORSV conserved gene sequences, including MN057680.1, HQ644131.1, MG821323.1, EU683879.1, MN027920.1, and so on, were obtained from NCBI Genbank. DNAMAN (Lynnon Biosoft, San Ramon, CA, USA) was used to analyze the sequences and identify the highly conserved regions of the gene sequence to be amplified. Subsequently, SnapGene (Genomic Sciences Laboratory, NC State University, Raleigh, NC, USA) software was used to design specific primers (Table 1) for CymMV (7 pairs) and ORSV (5 pairs). To enable the formation of a complex between recombinases and primers, RPA primers were designed with a length of 30–35 bp. RPA primers need to be devoid of extended guanine residues at the 5′ terminal, palindromes, secondary structures, and direct or inverted repeats. The design of the duplex RPA primers, i.e., both forward and reverse primers, must not overlap or be complementary to any other sets of forward and reverse primers. To verify specificity, the primers underwent nucleotide BLAST against the NCBI database to ensure the absence of results that highly match other viral sequences. Sangon Biotech Co., Ltd. (Shanghai, China) carried out the synthesis of the aforementioned primers.

### 2.4. RPA Primer Screening

To assess the efficacy and specificity of primer design, we screened primers via the RPA reaction. The ingredients and reaction conditions of the RPA were employed according to the specifications provided by the TwistAmp Basic Kit manufacturer (TwistDx, Maidenhead, SL6 4XE, UK). The prepared cDNA or plasmid was used as the template, and ddH_2_O served as the negative control. The mixture for the RPA reaction comprised 29.5 μL of rehydration buffer, 12.2 μL of nuclease-free water, 2.5 μL of magnesium acetate (280 mM), 2.4 μL (10 μM) of each primer, and 1 μL of cDNA or plasmid. Following the addition of the above solution, the reaction tube was mixed well, instantaneously centrifuged, and then incubated at 39 °C for 30 min. The RPA reaction product was obtained after terminating the reaction on ice. The RPA reaction product was combined with an equal volume of purified buffer (Tris saturated phenol:chloroform:isoamyl alcohol = 25:24:1). The solution was mixed well in a centrifuge tube and centrifuged at 12,000 rpm at 4 °C for 5 min to obtain the purified reaction solution. The purified product of 5 μL RPA was mixed with 0.5 μL loading buffer, and 2% agarose gel electrophoresis was conducted for 40 min to detect the product.

### 2.5. Construction of the Recombinant Plasmid Standard

Using viral cDNA as a template, RPA amplification and purification were performed with the above-screened primers. The 20 μL purified product was detected by 2% agarose gel electrophoresis, and the target fragments were purified again using the Takara Mini BEST Agarose Gel DNA Extraction Kit Ver. 4.0 (Code 9762) (Takara Biomedical Technology (Beijing) Co., Ltd., Beijing, China). The purified products were cloned and inserted into the pMD-19T vector, which resulted in the construction of two recombinant plasmids. The plasmids were extracted from bacterial suspensions containing accurate gene sequences using the Takara Mini BEST Plasmid Purification Kit Ver. 4.0 (Code 9760) (Takara Biomedical Technology (Beijing) Co., Ltd., Beijing, China). The constructed plasmids were digested with Hind III, and 5 μL of each product was extracted for agarose gel electrophoresis recovery. The resulting plasmid products were named pMD19T-CymMV and pMD19T-ORSV. The concentrations of the recombinant plasmids pMD19T-CymMV and pMD19T-ORSV were measured, and then the numbers of copies were calculated and serially diluted to 10^9^–10^0^ copies/μL.

### 2.6. Optimization of the Duplex RPA Method

The concentration of primers, temperature, and reaction time significantly affect the amplification effect in RPA reactions. To evaluate duplex RT-RPA reactions, positive plasmid mixtures were used, and ddH_2_O served as the negative control. The RPA reaction was performed with different amounts and proportions of CymMV and ORSV primers: 1.0 and 0.5 μL, 1.8 and 0.9 μL, 1.8 and 1.8 μL, and 1.8 and 2.4 μL. After determining the ideal ratio and quantity of primers, we proceeded to optimize the reaction temperature and time for duplex RPA. Subsequently, we conducted six rounds of tests to pinpoint the optimal temperature at which to carry out the reaction. The temperatures we tested were 30, 33, 36, 39, 42, and 45 °C. Next, we proceeded to optimize the reaction time, conducting tests at 15, 20, 25, 30, 35, and 40 min to determine the ideal duration.

### 2.7. Specificity Analysis

The specificity of detection for the duplex RPA assay was examined by employing samples infected with CymMV, ORSV, CymRSV, CGMMV, TMV, PLAMV, PVX, ToBSV, CMV, LMoV, CarMV single virus, and sample infected with both CymMV and ORSV, and ddH_2_O served as the negative control.

### 2.8. Sensitivity Analysis

The lower limits of detection for the duplex RPA assay were examined by employing the diluted mixed plasmids pMD19T-CymMV and pMD19T-ORSV (ranging from 10^9^ to 10^0^ copies) as templates, ddH_2_O served as the negative control. The reaction systems and procedures were consistent with the duplex RPA assay implemented in this research.

### 2.9. RPA Reliability for Field Samples

The duplex RPA method developed here was implemented to examine 20 field samples with suspected virus infection mentioned in 2.1.2, and ddH_2_O served as the negative control, and comparing the resulting RPA outcomes with past results of gene chip tests using the GeneTop Orchid^5^ RE Kit (GeneTop Biotech. (Taiwan) Co., Ltd., Taiwan, China).

## 3. Results

### 3.1. Screening of the Optimal Primer for the RPA Assay

**Figure 1:**
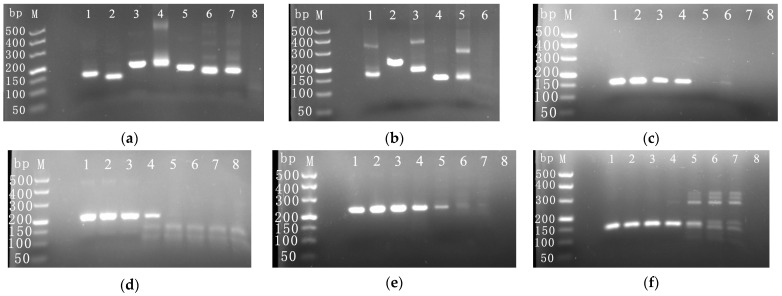
RPA detection of screening CymMV and ORSV primers. (**a**) Pictures of amplification with seven CymMV primers. Among them, 1–7 represent amplification products of primer pairs of CymMV-F1/CymMV-R1 to CymMV-F7/CymMV-R7, respectively, and 8 represented negative control. (**b**) Pictures of amplification with five ORSV primers. Among them, 1–5 represent amplification products of primer pairs of ORSV-F1/ORSV-R1 to ORSV-F5/ORSV-R5, respectively, and 6 represented negative control. (**c**) The sensitivity of primer pairs of CymMV-F2/CymMV-R2 was tested using cDNA as template. (**d**) The sensitivity of primer pairs of CymMV-F5/CymMV-R5 was tested using cDNA as template. (**e**) The sensitivity of primer pairs of ORSV-F2/ORSV-R2 was tested using cDNA as template. (**f**) The sensitivity of primer pairs of ORSV-F4/ORSV-R4 was tested using cDNA as template. In (**c**–**f**), the RNA concentrations of the cDNA reverse transcription template used were that 1–7 representing 10–10^−5^ ng/μL, respectively, and 8 represented negative control.

The RPA analysis demonstrated that all seven pairs of CymMV primers were able to amplify the target sequence (Figure 1a). Of these, primer pairs of CymMV-F2/CymMV-R2 and primer pairs of CymMV-F5/CymMV-R5 produced a single amplification band, while the other primers were associated with nonspecific amplification. All five pairs of ORSV primers were also found to amplify the target sequence (Figure 1b), with only primer pairs of ORSV-F2/ORSV-R2 and primer pairs of ORSV-F4/ORSV-R4 producing a single amplification band, while the other primers were associated with nonspecific amplification. Overall, the results indicate the successful amplification efficiency of the designed primers. The sensitivity of the CymMV and ORSV primers, each with a single amplification band, was determined (Figure 1c–f). The results indicated that primer pairs of CymMV-F2/CymMV-R2 of CymMV and primer pairs of ORSV-F2/ORSV-R2 of ORSV exhibited superior sensitivity and specificity compared to the other primers. Consequently, primer pairs of CymMV-F2/CymMV-R2 and primer pairs of ORSV-F2/ORSV-R2 were chosen for subsequent experiments.

### 3.2. Optimization of Duplex RPA Detection

#### 3.2.1. Optimization of Primer Proportions and Amounts

**Figure 2:**
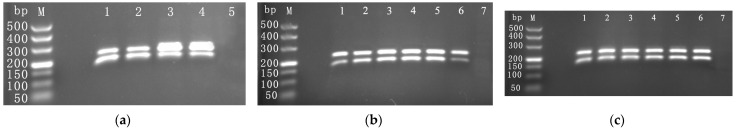
Optimization and establishment of duplex RPA assay. (**a**) Optimization of primer proportions and amounts of CymMV and ORSV. Among them, the primers’ addition amounts of 1–4 representing CymMV and ORSV were 1.0 μL and 0.5 μL, 1.8 μL and 0.9 μL, 1.8 μL and 1.8 μL, 1.8 μL and 2.4 μL, respectively, and 5 represented negative control. (**b**) Optimization of amplification temperature. Among them, 1–6 represent 30, 33, 36, 39, 42, and 45 °C, respectively, and 7 represents negative control. (**c**) Optimization of amplification time. Among them, 1–6 represent 15, 20, 25, 30, 35, 40 min, respectively, and 7 represents negative control.

The concentration and proportion of primers can impact the efficiency of multiple RPA amplifications [39]. The number of primers was optimized under the detection conditions of 39 °C for 30 min required by the kit. As revealed by the findings depicted in Figure 2a, when the primers’ amounts of CymMV and ORSV were, respectively, 1.8 and 1.8 μL or 1.8 and 2.4 μL, the brightness of the CymMV amplification band was relatively weak, and the ORSV amplification strip showed multiple bands. These outcomes were associated with different proportions and amounts of primers utilized for the detection of CymMV and ORSV primers. With 1.0 and 0.5 μL or 1.8 and 0.9 μL of the CymMV and ORSV primers, respectively, the amplification band of CymMV was relatively bright, there were no multiple bands in the ORSV amplification, and the bands of the two viruses were brighter overall with 1.0 and 0.5 μL of the CymMV and ORSV primers, respectively. Thus, the final primer addition amounts for CymMV and ORSV were determined to be 1.0 μL and 0.5 μL, respectively.

#### 3.2.2. Optimization of Amplification Temperature

The amplification temperature experiments for the duplex RPA revealed that the target bands of the two viruses could be amplified at 30–45 °C (Figure 2b). The bands for the two viruses were brightest at 39 °C, and the brightness of virus detection progressively decreased with increasing or decreasing temperatures. Thus, the duplex RPA’s optimal reaction temperature is 39 °C.

#### 3.2.3. Optimization of Amplification Time

The optimal amplification time experiments for the duplex RPA showed that (Figure 2c) the target bands of the two viruses could be clearly amplified at 15–40 min. The amplification bands for CymMV were weaker when the amplification time was 15–30 min, while the amplification bands for both viruses were brighter when the amplification time was 35–40 min. Therefore, considering amplification band clarity and time consumption, the optimal reaction time of duplex RPA was 35 min.

### 3.3. Establishment of the Duplex RPA Reaction

By optimizing the duplex RPA detection system and procedure, the final reaction system is that sample cDNA 1 μL, rehydration buffer 29.5 μL, 10 μM CymMV upstream and downstream primes 1.0 μL, 10 μM ORSV upstream and downstream primes 0.5 μL, nuclease-free water 14 μL, 280 mM magnesium acetate solution 2.5 μL. The reaction condition was 39 °C for 35 min.

### 3.4. Specific Detection

**Figure 3:**
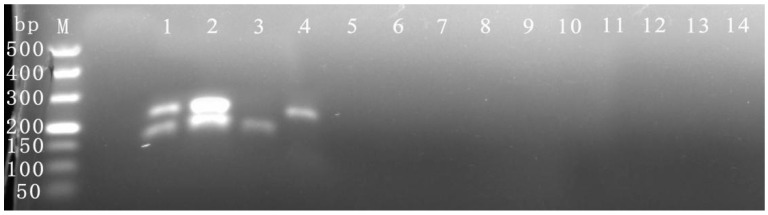
Specific detection of duplex RPA. Among them, 1 represents the positive plasmid carrying CymMV and ORSV, 2 represents the positive sample carrying both viruses of CymMV and ORSV, and 3–13 represent the positive sample carrying single virus of CymMV, ORSV, CymRSV, CGMMV, TMV, PLAMV, PVX, ToBSV, CMV, LMoV and CarMV, respectively, and 14 represents negative control.

The cDNA samples were tested for the presence of single viruses, including CymMV, ORSV, CymRSV, CGMMV, TMV, PLAMV, PVX, ToBSV, CMV, LMoV, and CarMV, as well as a sample containing both the CymMV and ORSV viruses, using the established duplex RPA detection system and program. These results (Figure 3) indicated that the established duplex RPA detection system can efficiently and precisely detect CymMV and ORSV without generating non-specific amplification of the remaining nine pathogens. Thus, the method can be considered highly specific.

### 3.5. Sensitivity Detection

**Figure 4:**
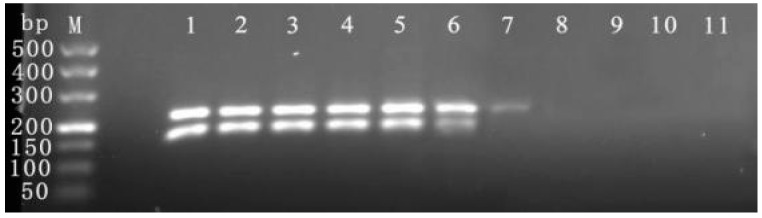
Sensitivity detection of duplex RPA. Among them, 1–10 represents 10^9^–10^0^ copies of pMD19T-CymMV and pMD19T-ORSV, and 11 represents negative control.

The established duplex RPA detection system and program were utilized to detect 10^9^–10^0^ copies of the plasmid standards to determine its sensitivity. The results of the detection indicated (Figure 4) that the detection limits of the established duplex RPA detection system for pMD19T-CymMV and pMD19T-ORSV were 10^4^ copies and 10^3^ copies, respectively. These results indicate that the duplex RPA detection system established by the invention has high sensitivity.

### 3.6. Sample Detection

**Figure 5:**
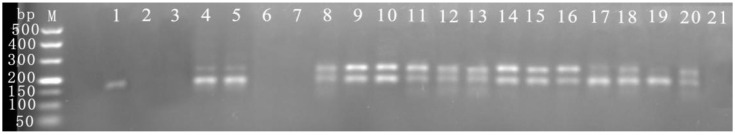
Sample detection of duplex RPA. Among them, 1–20 represent samples to be detected, and 21 represents negative control.

The duplex RPA detection system and protocol were utilized on 20 samples in the laboratory, and the resulting RPA detections were statistically assessed with the laboratory’s previous gene chip testing outcomes. The results indicate that in the duplex RPA assay, 80% (16/20) of the samples were found to carry CymMV, 70% (14/20) of samples carried ORSV; among them, 14 plants were dual-infected with CymMV and ORSV (Figure 5). In contrast, the gene chip method detected CymMV and ORSV in 40% (8/20) and 20% (4/20) of the samples, respectively; among them, two plants were dual-infected with CymMV and ORSV (Supplementary Table S1). These findings show that the detection system developed in this study enhances the detection rate of CymMV and ORSV by 100% and 250%, respectively, in comparison with the gene chip. Additionally, the sample analysis outcomes further confirm the precision and sensitivity of the newly introduced detection system.

## 4. Discussion

Viral disease, also known as “plant cancer”, is challenging to cure once it infects a plant. Each year, various crops experience varying degrees of production loss or even complete collapse due to viral infections. Therefore, preventing and controlling viral diseases has become a global concern. CymMV and ORSV, as the two most severe viruses that infect orchids, also infect over 20 species of plants [40]. This results in considerable economic and ornamental losses for orchids. Hence, identifying these viruses is of significant importance, particularly in the context of rapid testing. Multiple conventional techniques have been developed for detecting orchid viruses. Xie et al. conducted ELISAs to determine the virulence rates of CymMV and ORSV in various orchid cultivars [17]. Kim et al. developed a dual RT-PCR detection method for the simultaneous identification of CymMV and ORSV [18]. More recently, in 2022, Xu et al. established a reverse transcription loop-mediated isothermal amplification (RT-LAMP) technique for detecting CymMV and ORSV [1]. Sun developed a TaqMan probe-based triplex RT-qPCR protocol for detecting CymMV, ORSV, and CymRSV simultaneously [14]. Several of these assays typically involve costly equipment, extended durations, and rigorous operating specifications [41].

In recent years, RPA technology has gained widespread use due to its ability to complete amplification within 40 min at constant temperature. Previous reports have highlighted the successful application of the RPA method in detecting common human, animal, and plant pathogens. These reports include studies by Castellanos-Gonzalez et al. [42], Wang et al. [43], and Wu et al. [44]. Notably, Kim et al. [39] established an RPA detection system for CymMV in 2022. Studies have demonstrated the superior efficiency, time-saving capacity, and cost-effectiveness of RPA detection systems for the simultaneous detection of multiple pathogens compared to single RPA detection systems [19]. Nevertheless, there are currently no RPA detection systems capable of simultaneously detecting both CymMV and ORSV, which are the two most prevalent viruses causing severe infection in orchids.

In this study, we present a duplex RPA method that is both sensitive and specific in accurately identifying CymMV and ORSV. Primer specificity and concentration are critical factors influencing amplification efficiency during the RPA reaction [45]. To ensure specificity, primers were designed based on the highly conserved sequences of the CymMV and ORSV CP genes and screened accordingly. To enhance amplification efficiency, we investigated the effects of various sets of primer pairs of CymMV-F2/CymMV-R2 and primer pairs of ORSV-F2/ORSV-R2 concentrations. The optimal primer concentrations were 1.0 μL for CymMV and 0.5 μL for ORSV. Additionally, in comparison to traditional PCR detection, the detection system established in this study reduced the detection time from approximately 3 h to approximately 1 h. It also facilitates detection under constant temperature conditions of 39 °C, with minimal dependence on equipment and the capability to detect two different types of orchid viruses simultaneously. These factors have substantially enhanced the effectiveness and efficiency of detection. Specific detection of CymRSV, CGMMV, TMV, PLAMV, PVX, ToBSV, CMV, LMoV, and CarMV did not yield nonspecific amplification. This indicates that the detection system established in this study exhibits strong specificity. The detection limits for pMD19T-CymMV and pMD19T-ORSV were 104 and 103 copies, respectively. These findings demonstrate the high sensitivity of the detection system established in this study. By employing the detection system and gene chip method to analyze a set of 20 orchid samples, it has been discovered that the detection system constructed in this study enhances the rates of detection for CymMV and ORSV by 100% and 250% proportionately when compared to the gene chip method. Therefore, the duplex RT-RPA assay developed for CymMV and ORSV is a rapid and highly sensitive method for detecting orchid pathogens at rural inspection stations and other basic agricultural technology service points that lack high-precision temperature instruments.

## 5. Conclusions

This study established an RT-RPA assay for the swift, precise, and simultaneous detection of CymMV and ORSV in orchids. The RT-RPA duplex detection system developed in this study has low dependence on precision instruments such as thermal cycling apparatus, short time consumption, strong specificity, high sensitivity and practicality, as well as great potential for further development. This research method enables the rapid detection of field samples with a low threshold and can be widely applied in rural inspection stations and other basic agricultural technology service points. It effectively solves the problem of the ‘last mile’ in agricultural science and technology service. Additionally, it enhances the quality of orchid products and enterprise economic benefits, aiding in the healthy development of the orchid seed industry.

## Figures and Tables

**Table 1:** Primers designed for RPA detection of CymMV and ORSV.

Primer Name	Sequences (5′–3′)	Fragment Length (bp)
CymMV-F1	AATCTGATGCTGGCCACTAACGATCCGCCCGC	173
CymMV-R1	ATCGAGTGCGCAGCACGTTCACGGTCAGTAGGG	
CymMV-F2	AATTGTGGGTTAACAACCTTGGCCTCCCCGCCG	153
CymMV-R2	TAGAGCGGCGCGACGGACGTCAGGTTTCGTAGGG	
CymMV-F3	AAACCTGACGTCCGTCGCGCCGCTCTAGCC	226
CymMV-R3	ATTCAGCAGGTTCCAGTGCGGCAGTGGAATCG	
CymMV-F4	ACACAGTAGGTACCGCGGCCATTGACCTGGC	212
CymMV-R4	ATCGTTAGTGGCCAGCATCAGATTCCACACC	
CymMV-F5	AAGAGTGCTACCCTGCTCGGTTTCTGCCCTACG	200
CymMV-R5	AACCGAGTATCCTCCTGGAAACCAGCCTTGGC	
CymMV-F6	TTCCAGGAGGATACTCGGTTTGCCGCCTTTGAC	173
CymMV-R6	ATGAGGTTGCCGTTTTGGATACGCTGACGG	
CymMV-F7	TTCCACTGCCGCACTGGAACCTGCTGAATGGC	178
CymMV-R7	ATAGAGGGTGTTGGTGGAGCCAAGATGGCC	
ORSV-F1	TTCCTACTTTGACCAGTAGGTTCCCTGCAGGC	167
ORSV-R1	TAATGTTTCCGTAGTTGTCGGATTCTGCGG	
ORSV-F2	AATCAGTTCCAAACACAACAAGCTCGAACAACTG	224
ORSV-R2	TAGTTGTCGGATTCTGCGGATTTTCTACCTCG	
ORSV-F3	TGCAGGCGCTGGTTACTTCAGAGTTTATCGC	180
ORSV-R3	ATTGCTACAGTTGCATCATCAACTCTACGAG	
ORSV-F4	TAGAAAATCCGCAGAATCCGACAACTACGG	145
ORSV-R4	AATGAGACTTGATTGTACATACCAGTTCCC	
ORSV-F5	ATGCAACTCGTAGAGTTGATGATGCAACTGTAGC	142
ORSV-R5	TAGGAAGAGGTCCAAGTAAGTCCAGACATCG	

## Data Availability

The original contributions presented in the study are included in the article, further inquiries can be directed to the corresponding authors.

## Supplementary Materials

The following supporting information can be downloaded at: https://www.mdpi.com/article/10.3390/v16040543/s1, Table S1: Results of gene chip detection.
