# Supplementary material for: Development and Application of a Duplex RT-RPA Assay for the Simultaneous Detection of Cymbidium mosaic virus and Odontoglossum ringspot virus
Source: Viruses. 2024 Mar 30;16(4):543. doi: 10.3390/v16040543 (PMC11054353; doi:10.3390/v16040543)

Table S1. Results of gene chip detection

Number	Plant sources	Place of collection	Symptoms	Gene chip detection results		
				Picture of detection results	Detection virus	Detection results
1	*Dendrobium* '135'	Yunnan	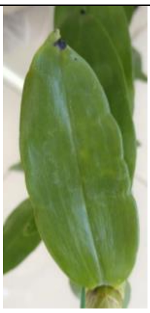	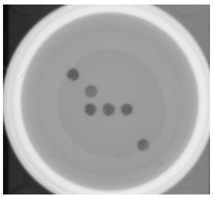	CymMV	(+)
					ORSV	(-)
2	*Oncidium* 'OB-2'	Yunnan	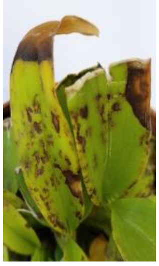	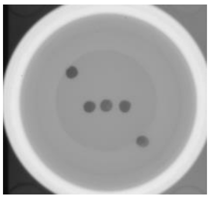	CymMV	(-)
					ORSV	(-)
3	*Zygopetulum* 'Zygopetalums'	Fujian	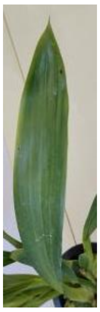	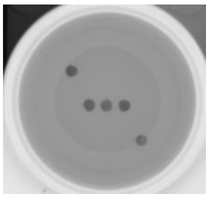	CymMV	(-)
					ORSV	(-)
4	*Zygopetulum* 'Zygopetalums'	Fujian	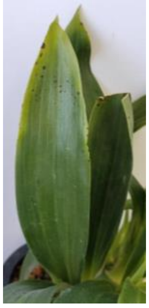	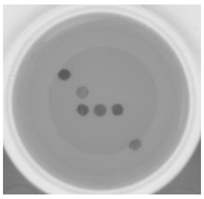	CymMV	(+)
					ORSV	(-)
5	*Zygopetulum* 'Zygopetalums'	Yunnan	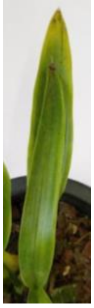	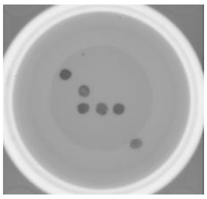	CymMV	(+)
					ORSV	(-)

6	*Oncidium* 'Sweet Sugar'	Guangxi	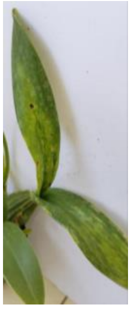	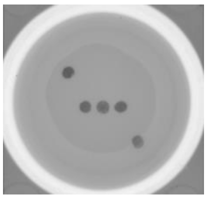	CymMV	(-)
					ORSV	(-)
7	*Oncidium* 'MXL'	Guangxi	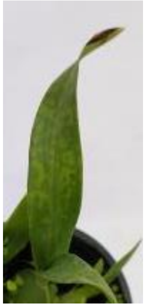	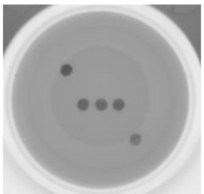	CymMV	(-)
					ORSV	(-)
8	*Phalaenopsis* 'amabilis'	Yunnan	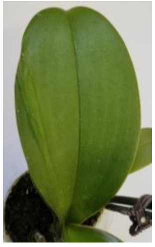	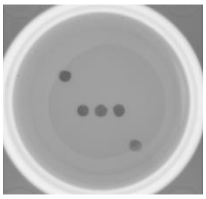	CymMV	(-)
					ORSV	(-)
9	*Phalaenopsis* 'Ruyi'	Yunnan	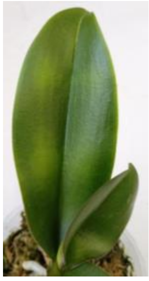	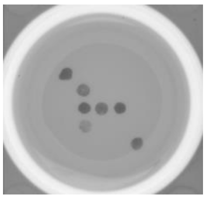	CymMV	(+)
					ORSV	(+)
10	*Phalaenopsis* 'Ruyi'	Yunnan	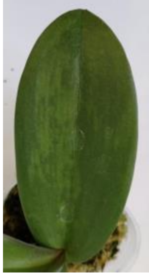	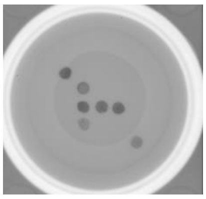	CymMV	(+)
					ORSV	(+)
11	*Phalaenopsis* 'innsbruck'	Guangxi			CymMV	(-)

			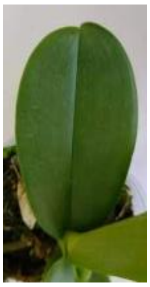	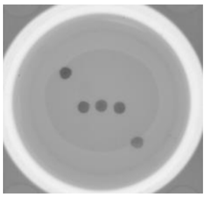	ORSV	(-)
12	*Phalaenopsis* 'Juguangdeng'	Guangxi	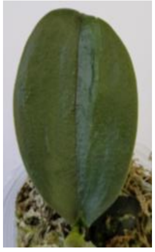	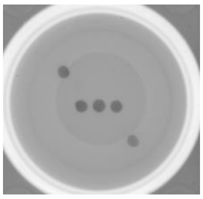	CymMV	(-)
					ORSV	(-)
13	*Phalaenopsis* 'Jinju'	Fujian	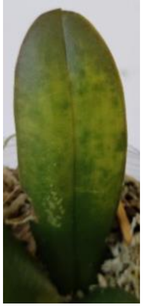	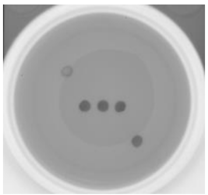	CymMV	(-)
					ORSV	(-)
14	*Phalaenopsis* 'Formosa Sunrise'	Fujian	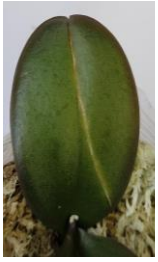	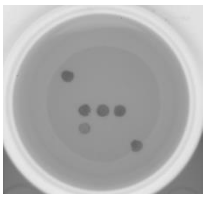	CymMV	(-)
					ORSV	(+)
15	*Phalaenopsis* 'Chocolate'	Guangxi	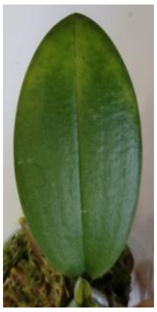	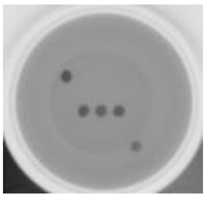	CymMV	(-)
					ORSV	(-)
16	*Phalaenopsis* 'Big chili'	Guangdo ng	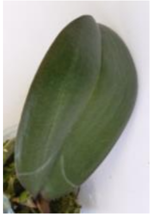	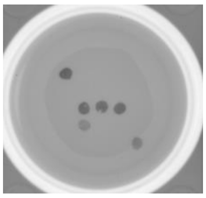	CymMV	(-)
					ORSV	(+)

17	*Zygopetulum* 'Zygopetalums'	Guangdong	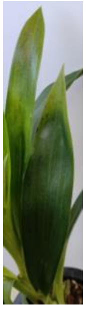	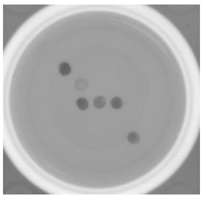	CymMV	（+）
					ORSV	（-）
18	*Dendrobium* '135'	Guangdong	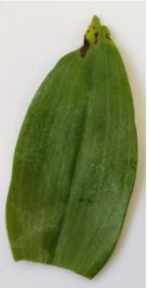	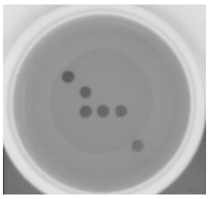	CymMV	（+）
					ORSV	（-）
19	*Zygopetulum* 'Zygopetalums'	Guangdong	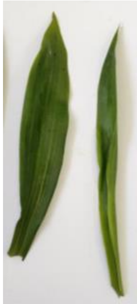	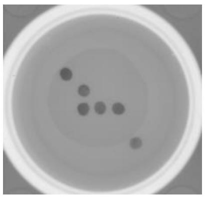	CymMV	（+）
					ORSV	（-）
20	*Phalaenopsis* 'Feilala'	Guangdong	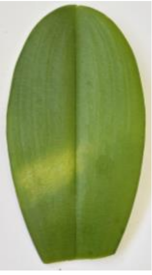	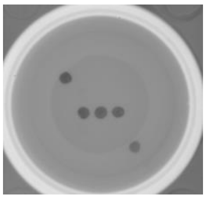	CymMV	（-）
					ORSV	（-）

Note :The test results of the gene chip are read as follows:

Negative sample: 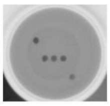 Samples carrying CymMV: 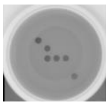

Samples carrying ORSV: 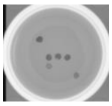 Samples carrying CymMV+ORSV: 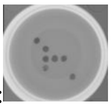

Supplement: Supplementary file 1 [file viruses-16-00543-s001.zip › viruses-2898762-supplementary.pdf]
